# The Impact of a Web-Based Restorative Dentistry Course on the Learning Outcomes of Dental Graduates: Pre-Experimental Study

**DOI:** 10.2196/51141

**Published:** 2024-03-05

**Authors:** Rasha Al-Sbei, Jawdat Ataya, Issam Jamous, Mayssoon Dashash

**Affiliations:** 1 Medical Education Program Syrian Virtual University Damascus Syrian Arab Republic; 2 Faculty of Dental Medicine Damascus University Damascus Syrian Arab Republic

**Keywords:** restorative dentistry, online learning, dental education, dental graduates, Syria, education, dental, dentistry, dental practice, effectiveness, educational program, survey

## Abstract

**Background:**

Restorative dentistry plays a crucial role in dental practice, necessitating professionals to stay abreast with the latest advancements in the field. The advancement of technology has made web-based learning a widely used method of education delivery in dentistry, providing learners with extensive information and flexibility.

**Objective:**

This study aims to evaluate how effective an online educational course in restorative dentistry is for dental graduates in Syria.

**Methods:**

This study used a pre-experimental study design, with pretest and posttest assessments to measure changes in participants’ knowledge and skills. A total of 21 dental graduates completed the online course in restorative dentistry, which was hosted on Moodle, using the learning management system of the Syrian Virtual University. Participants were provided with a suggested learning sequence and had the flexibility to navigate the course on their own and at their own pace. The course was developed based on the principles of web course design and web-based course development using the ADDIE (Analysis, Design, Development, Implementation, and Evaluation) general instructional design model. The pretest and posttest assessments consisted of 50 multiple-choice questions with a single correct answer, aligning with the course content. Furthermore, participants were asked to complete a course acceptance survey upon finishing the course.

**Results:**

The results showed a significant improvement in the participants’ knowledge of restorative dentistry, supported by a statistically significant *P* value of less than .05. The effect size of the difference between the pre and posttest indicated that the effect size, as indicated by ω^2^, demonstrated a significant 62.1% difference between the pre and posttest, indicating a high and statistically significant effect. Furthermore, the value derived from the Haridy obtained work ratio formula indicated that the educational program was effective, with an effectiveness amount of 3.36%. Additionally, 93% (n=19) of respondents expressed confidence in having gained the expected benefits from the educational course upon its completion.

**Conclusions:**

The findings indicated a notable enhancement in the participants’ understanding of restorative dentistry. The participants’ high satisfaction rate and positive feedback from the course acceptance survey further emphasize the favorable reception of the web-based learning approach. This study highlights the potential of web-based learning in dental education, opening the door for future research in this area. The findings of this study carry important implications for the design and implementation of web-based educational programs in dentistry, suggesting that such programs can serve as an effective tool for continuous professional development in the field.

## Introduction

Restorative dentistry is a crucial aspect of dental practice, involving the use of various materials and techniques to restore the function and aesthetic appearance of teeth [1]. Continuous education, particularly in restorative dentistry, is essential for dental graduates and practitioners to stay updated with the latest developments in the field [2-4].

In recent years, web-based learning, also known as e-learning, has gained popularity as a method of delivering education in dentistry, thanks to technological advancements [5,6]. This approach provides learners with access to a wealth of information while offering the flexibility to learn at their own pace, overcoming the limitations of time and space [7]. In response to this trend, the dental profession has developed web-based dental courses and web-based continuing dental education programs [8]. The global implementation of web-based learning in dentistry has been further accelerated by the COVID-19 pandemic [9,10].

The transition to new education delivery models, such as web-based education, has reshaped teaching methods into a blended learning methods, particularly in dental education [11,12]. Studies have shown that web-based teaching is perceived as equally as effective as traditional classroom approaches in terms of knowledge acquisition and academic performance [1,13]. Web-based dental education programs and courses have been developed to enhance the knowledge of dental students and practitioners, providing them with easy access to a wide range of information and fostering empowerment [14-16].

Several universities and dental schools worldwide have integrated web-based education into their dental programs, acknowledging its advantages and potential to enhance patient health care outcomes through a patient-centered approach and continuous learning [17-19]. However, while web-based learning offers numerous advantages, careful consideration of technical and pedagogical factors is essential to ensure its effectiveness. Hands-on training with manikins during preclinical education remains crucial [19-21].

This study aims to evaluate the effectiveness of a web-based educational course in restorative dentistry in improving the learning outcomes for Syrian dental graduates. Through assessing the influence of this web-based course, we aim to contribute to the understanding of the benefits and effectiveness of web-based learning in dental education.

## Methods

### Ethical Considerations

The ethical approval of this study was obtained from the Syrian Virtual University Ethics Committee (237/0) on January 7, 2023. This study involved a pre-experimental web-based course with human participants. The course did not involve any harmful interventions or interactions that could harm the participants. There is a preprint version of this study [22].

Before their participation, informed consent was obtained from all participants. The consent form, written in Arabic, the first language of all participants, provided a clear description of the research objectives and procedures. Participants were assured that their information would be securely stored and used solely for this research. To ensure confidentiality and privacy, the study data collected from participants were anonymized and protected with appropriate security measures. Access to the participants’ identities was restricted to only one of the researchers involved in the study.

The participation questionnaire was indirectly distributed to approximately 50 graduates, and a voluntary and random selection process was conducted to include participants. Out of the 50 participants contacted individually, a total of 21 participants willingly agreed to take part in the study. The selection of participants was not influenced by any specific criteria or biases, ensuring a representative sample for the research. A total of 21 dental graduates completed the online course in restorative dentistry (OCRD) which used a pre-experimental study design with pretest and posttest assessments.

### Overview

The course was asynchronous and hosted on Moodle, using the learning management system (LMS) of the Syrian Virtual University. Each participant had an account and full access to the course at any time. The course duration was 3 weeks, and it was available on LMS during this duration with full access for participants, it was divided into 4 thematic units with resources including documents, downloadable articles, presentations, short educational videos, examinations, and surveys.

The OCRD was developed by the principal researcher, RA, based on the principles of web course design [23,24], and web-based course development [25,26], using the ADDIE (Analysis, Design, Development, Implementation, and Evaluation) general instructional design model [27]. The course and its contents were reviewed by several experts from the Faculty of Dentistry at Damascus University.

The course primarily focused on theoretical aspects related to clinical topics. The course provided participants with a comprehensive understanding of various clinical topics within the field of dentistry. It aimed to enhance their theoretical knowledge and conceptual understanding of the principles and practices relevant to restorative dentistry. The course covered a wide range of theoretical aspects, including but not limited to dental materials, treatment planning, occlusion, tooth preparation, and aesthetic considerations. Participants were exposed to evidence-based theories, research findings, and best practices in the field. The course was theory about clinical topics and consisted of four main themes: (1) composite restoration including anterior and posterior composite restoration, (2) diastema closure, (3) direct composite veneer, and (4) tooth whitening.

In addition, we supported the course with clinical applications and clinical cases. The participants were given a suggested sequence for optimal learning but were allowed to access and navigate the course at their own pace. Participant contributions were reviewed daily to guide them in their studies and provide feedback. The daily feedback was given by the course tutor. Discussion of any issues related to the revised unit was also allowed, and any doubts were addressed. The study outcomes were based on the structure of the course, which was organized into units, lectures, and educational videos. Tools, methods, and resources were selected for use in the course, which included a set of presentations and related educational short videos.

The participants were informed about the pretest and posttest assessments that would be conducted as part of the study, and they were required to complete both assessments. The pretest was administered before the start of the course, while the posttest was conducted immediately after the course ended.

The study population comprised participants who had graduated from the Faculty of Dentistry. The invitation to the course was sent to a group of graduate students. A total of 21 participants agreed and accepted the invitation to participate in the course, and informed consent was obtained from those who accepted the invitation. The participants have been recruited according to the inclusion criteria including participants who had completed all restorative dentistry courses in their undergraduate studies, had access to a laptop, iPad, or smartphone, had good internet access, and had good knowledge of both Arabic and English, including medical terminology. Participants who did not complete the pretest were excluded from the study.

The pretest and posttest assessments were designed to measure the participants’ knowledge of restorative dentistry. The assessments were based on the course content. The assessments consisted of 50 multiple-choice questions with a single correct answer. The questions were designed to cover the 4 main themes of the course. Each multiple-choice question had 1 correct answer and 3 distractor options. A correct response received 2 points toward a total score of 100, while an incorrect response received zero points. The test was web-based, with limited time access. The duration of the test was 40 minutes.

In addition, participants were also asked to complete a course acceptance survey at the end of the course. The survey consisted of Likert scale questions and open-ended questions. The Likert scale questions were designed to measure the participant’s satisfaction with the course, the quality of the course content, and the effectiveness of the course in improving their knowledge of restorative dentistry. The open-ended questions allowed participants to provide additional feedback and comments about the course.

Data collected from the assessments were analyzed using SPSS (version 25.0; IBM). Descriptive statistics, including means and SDs, were calculated to describe the participants’ demographic characteristics and their pretest and posttest scores. The paired-sample 2-tailed *t* test was used to compare the mean scores of the pretest and posttest assessments. A *P* value of less than .05 was considered statistically significant.

## Results

### Overview

The study included 21 participants, of which 12 (57%) participants were female and 9 (43%) participants were male, with ages ranging from 23 to 30 years. Sixteen (76%) participants graduated from public universities and 5 (24%) participants graduated from private universities. The participants were distributed as follows: 12 (57%) participants were master candidates, 8 (38%) participants were general dentists, and 1 (5%) participant was a specialist. All participants had different years of experience in dental practice with 7 (33%) dentists having only 1 year of experience, 6 (29%) dentists having 2 years of experience, 6 (29%) dentists having 3 years of experience, and only 2 (10%) dentists having more than 3 years of experience.

A total of 21 participants completed the course, with course completion tracked by the LMS, and the time taken to complete the course ranged from 2 to 7 days. The pretest and posttest results were collected and stored in Google Forms and analyzed using SPSS. All 21 participants completed both tests without any significant lack of information. The change in knowledge between pretest and posttest results was analyzed using the paired-sample 2-tailed *t* test. A *P* value of less than .05 was considered significant. The *P* values, mean scores, and SDs for the precourse and postcourse tests for the entire test and each section are shown in Table 1.

**Table 1:** Results of paired-sample 2-tailed *t* test to test knowledge level change.

Unit and test		Score, mean (SD)	*P* value	*t* test (*df*)
**Entire test**			<.001	9.592 (21)
	Precourse	53.05 (6.2)		
	Postcourse	77.24 (11.8)		
**Composite restoration**			<.001	4.468 (21)
	Precourse	17.8 (1.9)		
	Postcourse	21.52 (2.8)		
**Diastema management**			<.001	6.664 (21)
	Precourse	11.33 (3.2)		
	Postcourse	17.14 (4.1)		
**Direct composite veneer**			<.001	8.087 (21)
	Precourse	14.38 (3.6)		
	Postcourse	20.3 (2.9)		
**Teeth whitening**			<.001	9.118 (21)
	Precourse	9.52 (3.6)		
	Postcourse	18.3 (4.6)		

The normal distribution was tested using the 1-sample Kolmogorov-Smirnov test, which showed that the distribution of the sample was standard. The paired-sample 2-tailed *t* test was then applied, revealing no statistically significant difference in the post-course test results of the participants, regardless of their age group, gender, academic status, type of university, or prior experience with online courses (*P*=.10, *P*=.06, *P*=.06, *P*=.10, and *P*=.65, respectively). The effect size of the difference between the pre and posttest was also calculated using ω^2^, and the effect size was found to be 62.1%, indicating a high and statistically significant effect.

Furthermore, the effectiveness of the educational program was evaluated using Haridy obtained work ratio formula [28], which showed that the program was effective, with an effectiveness amount of 3.36%.

After analyzing the data, we found that there was no statistically significant difference (with a *P* value>.05) in the postcourse test results of the participants, regardless of their age group, gender, academic status, type of university, or prior experience with web-based courses.

### Acceptance Questionnaire

The internal consistency of the user acceptance questionnaire items, as determined by Cronbach α, was demonstrated to be 0.924, indicating high reliability. In terms of user acceptance, all survey respondents (N=21) reported satisfaction with the web-based course when using a 5-point Likert scale. Moreover, the majority of survey respondents 93% (n=19) believed that they gained the expected benefit from the educational course after its completion.

The attendees showed the highest agreement with the well-employed materials and the enthusiasm of the instructor in presenting the web-based course, and the lowest agreement with the technical resources and communication methods required to attempt the web-based course as presented in Table 2.

**Table 2:** Results of statistical analysis of the user acceptance questionnaire.

Number	Questionnaire	Results, mean (SD)
1	Level of knowledge at the beginning of the course	2.86 (0.727)
2	Level of knowledge at the end of the course	3.86 (0.478)
3	The learning process was easy and clear	3.67 (0.658)
4	I had full control of the learning process	3.81 (0.512)
5	Time assumed for the learning process was sufficient	3.86 (0.478)
6	I had full decisions in the sequences of the learning process	3.95 (0.218)
7	The learning objectives of the course were listed.	4.38 (0.590)
8	The e-content was well organized	4.57 (0.507)
9	Loads of the educational process were affordable	4.14 (0.727)
10	The course was organized to allow participants to participate adequately	4.29 (0.784)
11	The e-content was useful and gave added value	4.52 (0.512)
12	The e-content was obvious and clear	4.52 (0.512)
13	I think e-Learning is interesting	4.19 (0.512)
14	I believe that e-Learning is useful and gives an added value	4.14 (0.727)
15	I believe that the e-Learning process is easy and feasible	4.19 (0.814)
16	Log-in to the platform was easy and clear	4.38 (0.669)
17	Navigation with platform was easy and clear	4.48 (0.512)
18	The e-content on the platform was well-organized	4.62 (0.498)
19	The course instructor was active	4.43 (0.507)
20	The presentations were well-organized and clear	4.62 (0.498)
21	The recorded presentations were clear and understandable	4.19 (0.512)
22	The suggested educational videos were useful and related	4.33 (0.577)
23	The course instructor was available and useful	4.52 (0.512)
24	The feedback was direct and constructive	4.43 (0.507)

## Discussion

### Principal Findings

This study aimed to evaluate the effectiveness of a web-based educational program in restorative dentistry for increasing the knowledge of Syrian dental graduates. The course was designed based on the fundamental principles of web course design and web-based course development. A total of 21 participants completed the entire course. The results of the study showed a significant increase in the participants’ levels of restorative dentistry knowledge after completing the web-based course, as demonstrated by the postcourse test scores.

The course was also well-received by the participants, as indicated by their positive evaluations. These findings are consistent with previous studies that have investigated the effectiveness of web-based learning in enhancing participants’ knowledge and skills. This success of the course could be attributed to the well-structured presentation and content of the course, as well as the ease of use of the web-based platform (LMS-Moodle) and its flexibility in navigating the course content [29,30].

The highest score in the students’ precourse tests was obtained about composite restoration (first unit), which may be attributed to the fact that composite restoration is widely used and considered the most popular restorative material in dentistry. In contrast, the lowest score in the precourse test was obtained for tooth whitening (fourth unit), because tooth whitening lectures are only given in advanced dental practice programs and postgraduate studies. However, the students’ scores on specific knowledge areas in the postcourse tests showed a notable improvement in all units, including tooth whitening, despite the widespread use of tooth whitening in dental clinics to restore the aesthetic appearance of teeth [31]. This result ensures that the course has been focused on new concepts and interesting ideas that the participants had not been taught before, as well as influencing their daily practice.

The result of this study is consistent with those of Morales‐Pérez et al [29], Absi et al [32], and Rosenberg et al [33] regarding the effectiveness of web-based learning and web-based courses in increasing the knowledge of participants.

The current situation in Syria makes attending traditional courses challenging, due to, for example, the lack of transportation, fuel, and electricity; lack of sufficient and qualified places to hold the courses; and shortage of human resources and staff [34-36].

However, the course offered flexibility in terms of delivery and content review, allowing participants access to the platform 24 hours a day. This approach is similar to previous studies by Murphy et al [37] and Rosenberg et al [33]. Asynchronous web-based learning was also an option that enabled participants to revisit the course materials as needed, based on their commitments and social life as noted by Ruiz et al [38].

Asynchronous web-based learning is considered an attractive, flexible, and convenient option for learners, with lower costs and easy access to information. Similarly, Kenjrawi and Dashash [39] found that asynchronous electronic medical education is an effective and feasible approach for improving the knowledge and attitude of Syrian clinical practitioners [39].

The web-based course proved a valuable option for continuing medical education in Syria given the current circumstances in Syria and after the impact of the COVID-19 pandemic [40]. In dental education, computerized sources of information and virtual reality are increasingly being used as educational tools and have shown promise in training dental students [41]. These technological advancements have the potential to revolutionize dental education and enhance the learning experience of students.

The results of this study indicate that the participants expressed high levels of acceptance and satisfaction with the restorative dentistry course, as evidenced by the questionnaire results. The majority of the cohort (19/21, 93%) reported that the course provided the desired benefit, and all participants reported enjoying it. The well-designed course materials and the instructor’s enthusiasm for delivering the web-based course were also praised by participants. However, there were some areas of the course that needed improvement, such as technical resources and communication methods required to improve the web-based course.

To further assess the effectiveness of the web-based course, future research using larger sample sizes would be beneficial, to evaluate both knowledge and skills in each unit of restorative dentistry separately. Nevertheless, implementing web-based courses of restorative dentistry in continuous medical education programs in Syria is advisable, as it may help dentists stay up to date with minimal requirements.

### Limitations

The study did not assess the long-term retention of knowledge or the impact of this web-based course on the participants’ clinical practice. Furthermore, the study focused only on an OCRD. The findings might not apply to other areas of dentistry or other forms of web-based learning.

### Conclusion

This study provides evidence that web-based learning can be an effective tool in improving the knowledge and skills of dental graduates about restorative dentistry. The high satisfaction rate expressed by the participants and the positive feedback received through the course acceptance survey indicate that the web-based educational program was well-received by the participants. These findings support the potential of web-based learning in dental education and suggest that it can be a valuable tool for providing continuous education to dental professionals, as it can help dental professionals stay up to date with the latest advancements in their field, which can ultimately benefit the patients they serve.

## Abbreviations

ADDIE: Analysis, Design, Development, Implementation, and Evaluation
LMS: learning management system
OCRD: online course in restorative dentistry

## Data Availability

The data sets generated or analyzed during this study are available from the corresponding author on reasonable request.
